# Mitofusin 2 Promotes Apoptosis of CD4^+^ T Cells by Inhibiting Autophagy in Sepsis

**DOI:** 10.1155/2017/4926205

**Published:** 2017-11-19

**Authors:** Lan Ying, Guang-Ju Zhao, You Wu, He-Liang Ke, Guang-Liang Hong, Hui Zhang, Ning Dong, Yao Wu, Yong-Ming Yao, Zhong-Qiu Lu

**Affiliations:** ^1^Emergency Department, The First Affiliated Hospital of Wenzhou Medical University, Wenzhou 325000, China; ^2^Wenzhou Municipal Key Laboratory of Emergency, Critical Care, and Disaster Medicine, The First Affiliated Hospital of Wenzhou Medical University, Wenzhou 325000, China; ^3^Department of Emergency Medicine, The Second Affiliated Hospital of Zhejiang University School of Medicine, Hangzhou, Zhejiang 310009, China; ^4^Trauma Research Center, First Hospital Affiliated to the Chinese PLA General Hospital, Beijing 100048, China; ^5^College of Nursing, Wenzhou Medical University, Wenzhou 325000, China

## Abstract

Apoptosis of CD4^+^ T cells is a primary pathophysiological mechanism of immune dysfunction in the pathogenesis of sepsis. Mitofusin 2 (Mfn2), an integral mitochondrial outer membrane protein, has been confirmed to be associated with cellular metabolism, proliferation, and apoptosis. The function of Mfn2 in CD4^+^ T cell apoptosis in sepsis is poorly understood. Here, we discovered increased *in vivo* Mfn2 expression, autophagy deficiency, and elevated cell apoptosis in murine splenic CD4^+^ T cells after cecal ligation and puncture (CLP). We also observed almost identical results in splenic CD4^+^ T cells upon lipopolysaccharide (LPS) stimulation *in vitro*. Furthermore, overexpression of Mfn2 resulted in impaired autophagy and increased apoptosis in Jurkat cells. Pharmacological inhibition of autophagy with 3-methyladenine enhanced Mfn2 overexpression-induced cell apoptosis. In addition, overexpression of Mfn2 downregulated phorbol myristate acetate (PMA)/ionomycin-, rapamycin- and starvation-induced autophagy in Jurkat T cells. Taken together, these data indicate a critical role of Mfn2 in CD4^+^ T cell apoptosis in sepsis and the underlying mechanism of autophagy deficiency.

## 1. Introduction

Sepsis is defined as a life-threatening organ dysfunction caused by a dysregulated host response to infection [1]. Sepsis, a condition characterized by dysregulated inflammation [1] and immunity [2], has been a critical problem in the intensive care unit for decades. CD4^+^ T cell apoptosis is a primary mechanism in the pathophysiology of immune paralysis [3, 4]. The clinical outcomes of interventions designed to prevent apoptosis in sepsis have been disappointing; therefore, the underlying mechanisms need to be investigated further.

Mitofusin 2 (Mfn2), which resides in the outer membrane of mitochondria, plays a pivotal role in the fusion process of the outer mitochondrial membrane and in additional fusion-independent roles [5, 6]. The function of Mfn2 in apoptosis is stimulation and cell type dependent or tissue dependent. Studies have demonstrated the antiapoptotic function of Mfn2 in ovarian tissue [7], in the heart after ischemia/reperfusion injury [8] and in excitotoxicity of neurons [9]. In addition, the proapoptotic effect of Mfn2 was documented in hepatocellular carcinoma [10] and stress-induced death of heart muscle [11]. Moreover, data from our previous study showed the involvement of Mfn2 in high mobility group box-1 protein- (HMGB1-) induced immune dysregulation of CD4^+^ T cells [12] and in apoptosis of HMGB1-activated T cells [13]. Although there are a number of studies indicating that Mfn2 is associated with T cell apoptosis in sepsis, the potential role and underlying mechanism of Mfn2 in T cell apoptosis resulting from sepsis are not fully characterized.

Autophagy, a cellular degradation system distinct from the ubiquitin-proteasome system, is a primary mechanism for maintaining cellular homeostasis. Increasing evidence has shown a relationship between autophagy and apoptosis [14–19]. Moreover, studies have established that autophagy plays a key role in T cells [20, 21]. Recently, a study has shown that autophagy deficiency enhanced apoptosis in T cells after cecal ligation and puncture (CLP) [22]. In addition, several studies have revealed that Mfn2 can regulate autophagy both in the early initiation [23] and the late maturation process [24]. Hence, we hypothesized that Mfn2 might be involved in the regulation of CD4^+^ T cell apoptosis in sepsis and autophagy might be the potential mechanism.

Therefore, to evaluate the relationship between Mfn2 expression, autophagy level, and apoptosis of CD4^+^ T cells in sepsis, we performed *in vivo* and *in vitro* investigations. In addition, we constructed lentiviral vectors to up- or downregulate Mfn2 expression in Jurkat T cells to establish the effect of Mfn2 on autophagy level and cell apoptosis. Then, to identify the potential mechanism, we performed pharmacological intervention against autophagy.

## 2. Materials and Methods

### 2.1. Animals and Ethics Statement

BALB/c mice (male, 6–8 weeks old, 20 ± 2 g), obtained from the Laboratory Animal Center, Chinese Academy of Medical Sciences, Beijing, China, were used in these experiments. All experimental manipulations were performed in strict accordance with the National Institutes of Health Guide for the Care and Use of Laboratory Animals, with the approval of the Scientific Investigation Board of the Chinese PLA General Hospital (number SYXK2012-0014), Beijing, China.

### 2.2. Cell Line

The Jurkat T cell line was obtained from the Cell Resource Center of Shanghai Institutes of Biological Sciences (Shanghai, China) and was cultured in Roswell Park Memorial Institute- (RPMI-) 1640 medium supplemented with 10% fetal bovine serum in a humidified atmosphere of 5% CO_2_ at 37°C. In each experiment, we used Trypan blue exclusion to determine cell viability.

### 2.3. Medium and Reagents

The CD4^+^ T Cell Isolation Kit was obtained from Miltenyi Biotec GmbH, Bergisch Gladbach, Germany. Reagents, including LPS from *Escherichia coli* 0111:B4, 3-methyladenine, phorbol myristate acetate (PMA), and ionomycin, were purchased from Sigma-Aldrich, St. Louis, MO. The fluorescein (FITC) Annexin-V Apoptosis Detection Kit I was obtained from BD/PharMingen, San Diego, CA, and a One Step TUNEL Apoptosis Assay Kit was purchased from Beyotime Biotechnology, Shanghai, China. Antibodies, including anti-Mfn2, anti-LC3B, anti-Beclin1, anti-p62, anti-*β*-actin, and peroxidase- (HRP-) conjugated goat anti-rabbit secondary antibodies, were purchased from Cell Signaling Technology, Danvers, MA. Digitonin was purchased from Abcam, Cambridge, MA. Fluorescent antibodies, including FITC-conjugated affinipure goat anti-rabbit IgG and Cy™3-conjugated affinipure goat anti-rabbit IgG, were purchased from Jackson ImmunoResearch Laboratories, West Grove, PA. The Cyto-ID® Autophagy detection kit was purchased from Enzo Life Sciences, Raamsdonksveer, The Netherlands. Bafilomycin A1, rapamycin, and FlowCellect™ Autophagy LC3 Antibody-based Assay kits (FITC) were purchased from Merck Millipore, Billerica, MA.

### 2.4. Animal Sepsis Model

After being given water but not food overnight, mice were anesthetized with 0.3% pentobarbital sodium. The exposed cecum was ligated in the middle and punctured with a 22 G needle in the middle. Some feces were pressed out to ensure patency of the puncture. Then, the bowel loops were returned, and the abdominal wall was closed. Postoperatively, 1 ml of 0.9% saline was administered subcutaneously. In the sham group, mice were subjected to an identical operation, except the cecum was not ligated or punctured. In the first 6 hours, the survival rate was 100%, and the mice appeared to exhibit depression and diarrhea, which are typical symptoms and suggested successful construction of the animal sepsis model. The mortality rate 72 hours after CLP was approximately 50%–70%.

### 2.5. Isolation of Splenic CD4^+^ T Cells

The isolated spleens were washed with cold PBS twice and gently ground with a 400-mesh sieve to produce a single-cell suspension. Mononuclear cells were obtained with the use of Ficoll-Paque density gradient centrifugation, and then CD4^+^ T cells were separated by negative selection using CD4 microbeads according to the manufacturer's instructions. Briefly, mononuclear cells were incubated with biotin-antibody cocktail (10 *μ*l/10^7^ total cells) for 10 minutes at 4°C. They were then magnetically labeled with antibiotin microbeads (20 *μ*l/10^7^ total cells) for 15 minutes at 4°C and harvested through a negative selection MS column (>95% purity).

### 2.6. Lentiviral Generation and Transduction

Lentiviral vectors to induce overexpression of Mfn2 or shRNA specific to Mfn2 to silence Mfn2 were constructed by Shanghai GeneChem Co. Ltd. (Shanghai, China). A lentiviral vector that enveloped mCherry or RFP alone was also generated as a scramble control. The shRNA-Mfn2 sequence was 5′-AGGTTTACTGCGAGGAAAT-3′. Transfection was performed according to the manufacturer's instructions in lentiviral vector particle. The cells were seeded in 24-well plates at a concentration 2 × 10^6^/ml, and the appropriate number of lentiviral vectors equal to a multiplicity of infection (MOI) of 50 was added to the 24-well plates. After 12 hours, fresh RPMI-1640 (10% FBS) was used to replace the medium. For an additional 72 hours, the cells were observed with a fluorescence microscope to assess the transfection efficiency. In addition, Western blotting analysis was used to measure the transfection efficiency.

### 2.7. Experimental Design

#### 2.7.1. *In Vivo* Experiment

Sepsis mouse models were constructed by CLP, and then, mice were divided into three groups: the sham group, the CLP1D group, and the CLP3D group. After the indicated number of days, mice were sacrificed and splenic CD4^+^ T cells were isolated. Then, Mfn2 expression, autophagy level, and cell apoptosis were determined.

#### 2.7.2. *In Vitro* Experiment

Splenic CD4^+^ T cells, obtained from BALB/c mice, were cultured with LPS (10, 100, and 1000 ng/ml) or PBS for 24 hours. After stimulation, Mfn2 expression, autophagy level, and cell apoptosis were examined.

#### 2.7.3. Transfection Experiment

Jurkat T cells were transfected with lentiviral vector as described above and divided into 5 groups: the control group, the LV-Mfn2 group, the LV-mCherry group, the LV-Mfn2 RNAi group, and the LV-RFP group. After an additional 72 hours, cells were cocultured with or without one of the autophagy inducers, PMA (50 ng/ml)/ionomycin (1 *μ*M), rapamycin (100 nM), and starvation (low serum (<1% FBS) in RPMI-1640), for 24 hours. Subsequently, cells were collected to measure the autophagy level and cell apoptosis. In the autophagy intervention experiment, cells from the control group, the LV-Mfn2 group, or the LV-mCherry group were divided into three groups and subjected to the following treatments: DMSO for 24 hours, rapamycin (100 nM) for 24 hours, or 3-MA (10 mM) for 18 hours. After the indicated time, cells were harvested to evaluate autophagy level and cell apoptosis.

### 2.8. Flow Cytometry Analysis

Flow cytometry analysis was performed using a FACScan flow cytometer (BD Biosciences, Mountain View, CA). Approximately 10,000 events were analyzed from each sample. Data were acquired using CellQuest software.

### 2.9. Annexin-V Staining

Cell apoptosis was measured with Annexin-V-FITC plus 7-AAD according to the manufacturer's instructions. Briefly, cells were resuspended in 100 *μ*l binding buffer containing 5 *μ*l Annexin-V and 5 *μ*l 7-AAD and incubated for 15 minutes at room temperature, following washed by cold PBS twice. Subsequently, cells were diluted by adding 300 *μ*l of binding buffer and analyzed using flow cytometry within 1 hour. Data are shown as the percentage of Annexin-V-positive cells.

### 2.10. TUNEL Staining

Cell apoptosis was determined with a terminal deoxynucleotidyl transferase- (TdT-) mediated dUTP nick end labeling (TUNEL) assay kit according to the manufacturer's instructions. In brief, cells were fixed with 4% paraformaldehyde at room temperature for 1 hour and washed once with PBS. Then, cells were resuspended with 0.1% Triton X-100 at 4°C for 2 minutes and washed twice with PBS. The cells were then incubated with 50 *μ*l of TUNEL reaction mixture containing TdT and fluorescein-dUTP at 37°C for 1 hour. After incubation, cells were washed twice with PBS. The cells were resuspended in 300 *μ*l of PBS and analyzed using flow cytometry. Data are shown as the percentage of TUNEL-positive cells.

### 2.11. Membrane-Associated LC3-II Measured with Flow Cytometry

After being collected, cells were permeabilized with 0.25% digitonin at 4°C for 5 minutes and washed with PBS five times. Subsequently, cells were fixed with 4% paraformaldehyde at room temperature for 30 minutes and then washed with PBS three times. Cells were incubated with FITC-tagged LC3 antibody (1 : 1000) overnight at 4°C and then washed with PBS three times. Lastly, cells were resuspended in 400 *μ*l of PBS and analyzed with flow cytometry. Data are shown as the percentage of LC3-II-positive cells.

### 2.12. Cyto-ID Green Staining

Autophagosomes and autolysosomes were assessed with a Cyto-ID Autophagy detection kit according to the manufacturer's instructions. In brief, the harvested cells were washed once with assay buffer. Then, cells were resuspended in 250 *μ*l of assay buffer and stained by adding 250 *μ*l of diluted Cyto-ID green at 37°C for 30 minutes. After incubation, cells were centrifuged and then resuspended in 250 *μ*l of assay buffer, followed by flow cytometry analysis. Data are shown as the percentage of Cyto-ID green staining.

### 2.13. Western Blotting Analysis

After being collected, cells were lysed with lysis buffer on ice for 30 minutes and then centrifuged at 14,000 rpm for 30 minutes at 4°C. The protein concentration within the supernatants was detected using the bicinchoninic acid (BCA) method. Then, 22 *μ*g of total protein was mixed with appropriate SDS-loading buffer and then boiled at 92°C for 5 minutes. The proteins were separated in 8%–12% Tris-HCl sodium dodecyl sulfate-polyacrylamide gels (Pulilai Co., Beijing, China) and subsequently transferred to polyvinylidene fluoride (PVDF) membranes (0.45 *μ*m; Merck Millipore, Billerica, MA) by electroblotting. After being blocked with 10% skim milk at room temperature for 4 hours, the membranes were incubated with anti-Mfn2 (1 : 1000), anti-LC3B (1 : 1000), anti-Beclin1 (1 : 1000), anti-p62 (1 : 1000), and anti-*β*-actin (1 : 1000) antibodies overnight at 4°C. Subsequently, the members were incubated with horseradish peroxidase- (HRP-) conjugated goat anti-rabbit secondary antibody (1 : 5000) at room temperature for 1 hour. After being washed, the membranes were analyzed by adding ECL. The graph was analyzed using Quantity One software. The membranes could be recycled and used three times.

### 2.14. Laser Scanning Confocal Microscopy (LSCM)

Purified cells were resuspended with 4% paraformaldehyde at room temperature for 30 minutes and then permeabilized with 0.2% Triton X-100 at room temperature for another 20 minutes. Then, 5% bovine serum albumin (BSA) was used to block cells for 30 minutes at room temperature. Subsequently, cells were incubated with anti-LC3B (1 : 400) overnight at 4°C, followed by FITC-conjugated affinipure goat anti-rabbit IgG (1 : 100) or Cy3-conjugated affinipure goat anti-rabbit IgG (1 : 400) at room temperature for 1 hour. After being washed, the nuclei were stained with 4′,6-diamidino-2-phenylindole (DAPI) for 5 minutes. The cells were observed with laser scanning confocal microscopy.

### 2.15. Transmission Electron Microscopy

Purified CD4^+^ T lymphocytes were fixed with glutaraldehyde and stored at 4°C overnight. Following a series of manipulations, including fixation, dehydration, embedding, curing, biopsy, and dyeing, transmission electron microscopy was used to observe autophagic vacuoles that were double- or single-membrane structures containing digested cytoplasmic components or organelles.

### 2.16. Statistical Analysis

All data from three independent experiments are shown as the mean ± SD. One-way analysis of variance (ANOVA) was performed for statistical analysis. Fisher's least significant difference (LSD) was used to analyze comparisons between two groups. Differences with *P* values < 0.05 were considered statistically significant.

## 3. Results

### 3.1. Increased Mfn2 Expression, Autophagy Deficiency, and Upregulation of Cell Apoptosis in Murine Splenic CD4^+^ T Cells after CLP

**Figure 1:**
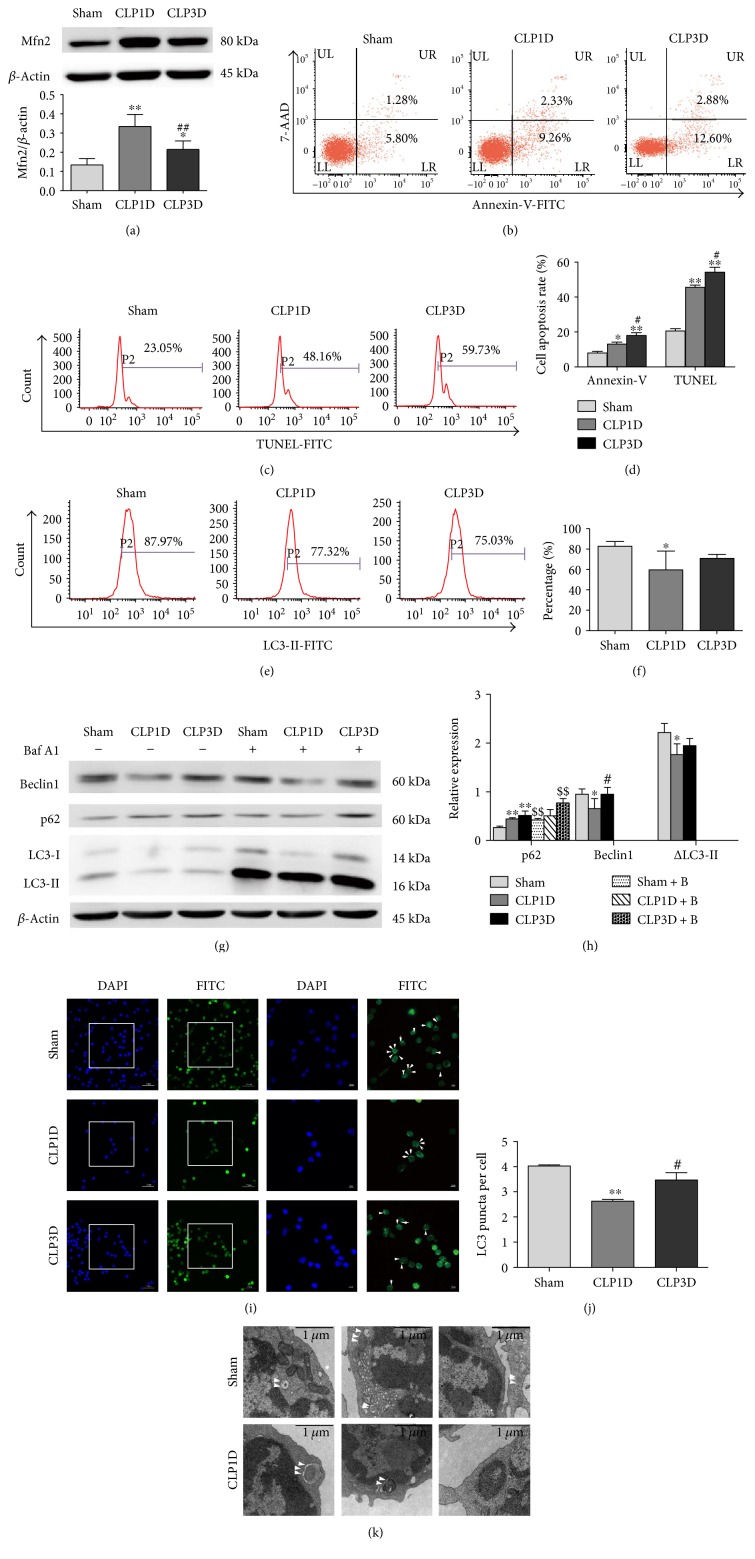
Increased Mfn2 expression, autophagy deficiency, and upregulated apoptosis in murine splenic CD4^+^ T cells after CLP. Splenic CD4^+^ T cells were isolated from mice 24 and 72 hours after CLP. (a) Representative immunoblots and densitometric values of Mfn2 are shown; *β*-actin served as an internal control. (b) Representative dot plots of Annexin-V staining. (c) Representative histograms of TUNEL staining. (d) Percentage of Annexin-V- or TUNEL-positive cells. (e, f) Representative histograms and total percentage of LC3-II-positive cells. (g, h) Isolated CD4^+^ T cells were cultured with or without bafilomycin A1 (0.1 *μ*M) for 4 hours, and the whole cell lysate was used to measure the protein expression of Beclin1, p62, and LC3-II. *β*-Actin served as an internal control. (i) Representative images of immunofluorescence staining against LC3. DAPI and FITC were used to label nuclei and endogenous LC3, respectively. The arrows indicate LC3 puncta. (j) Quantification of the average number of LC3 puncta per cell (*n* = 3 wells, 3 independent experiments, and >50 cells examined per experiment). (k) Transmission electron microscopy was used to observe autophagic vacuoles. The arrows indicate autophagic vacuoles. Data from three independent experiments are presented as the mean ± SD in the bar graph. Baf A1 or B: bafilomycin A1. ^∗^*P* < 0.05, significant difference versus the sham group or the sham + B group. ^∗∗^*P* < 0.01, significant difference versus the sham group or the sham + B group. ^#^*P* < 0.05, significant difference versus the CLP1D group. ^##^*P* < 0.01, significant difference versus the CLP1D group. ^$$^*P* < 0.01, significant difference versus the corresponding group without bafilomycin A1 treatment.

In this study, a CLP model was employed as an *in vivo* animal sepsis model. To determine the relationship between Mfn2, autophagy, and apoptosis in splenic CD4^+^ T cells, we monitored dynamic changes in these parameters after CLP. We chose 24 hours and 72 hours after CLP as the assessment time points. The expression of Mfn2 was significantly increased in murine splenic CD4^+^ T cells in the CLP group compared to that in the sham group, especially in the CLP1D group (Figure 1(a)). To determine the percentage of apoptotic cells, splenic CD4^+^ T cells isolated from septic mice were stained with both Annexin-V and TUNEL and then analyzed with flow cytometry. As shown in Figures 1(b), 1(c), and 1(d), the number of apoptotic cells (Annexin-V positive or TUNEL positive) was significantly increased in the CLP1D group in comparison to the sham group and remained elevated for up to 3 days (CLP3D).

Given that autophagy is a dynamic process, we evaluated autophagy formation and autophagic flux. Autophagosome formation was evaluated by monitoring the expression of Beclin1 and LC3-II using fluorescent puncta visualization after staining with immunofluorescent antibodies against endogenous LC3 using confocal laser scanning microscopy and autophagic vacuole observation using transmission electron microscopy (TEM). In addition, flow cytometry analysis was used to detect membrane-associated LC3-II based on the idea that permeabilization prior to fixation leads to extraction of the membrane-unbound LC3-I form. Autophagic flux was measured by examining the expression of p62 and immunoblots to monitor difference in the amount of LC3-II between samples in the presence and absence of inhibitor of lysosomal proteolysis. As shown in Figures 1(e) and 1(f), the expression of LC3-II measured with flow cytometry was significantly decreased in the CLP1D group but restored in the CLP3D group compared to that in the sham group. Considering the low expression of LC3-I, we used *β*-actin as the loading control to represent the relative expression of LC3-II. △LC3-II, the difference in the amount of LC3-II in the presence and absence of bafilomycin A1, was significantly reduced in the CLP1D groups compared to that in the sham group, and the expression of Beclin1, the first mammalian protein shown to play a critical role in initiation of autophagy, was decreased in the CLP1D group but returned to baseline in the CLP3D group (Figures 1(g) and 1(h)). Bafilomycin A1, an autophagosome-lysosome fusion inhibitor, increased LC3-II expression in groups with or without CLP and the p62 levels in the sham group and the CLP3D group, which exclude the possibility of impaired autophagic degradation (Figures 1(g) and 1(h)). As shown in Figures 1(i) and 1(j), a decreased number of LC3 puncta per cell were observed in the CLP1D group compared to that in the sham group. Moreover, we employed TEM for qualitative characterization of autophagy (Figure 1(k)). These observations indicated that autophagy was deficient at 24 hours and restored at 72 hours after CLP.

Taken together, these results showed enhanced Mfn2 expression, downregulated autophagy, and elevated apoptosis in CD4^+^ T cells 24 hours after CLP.

### 3.2. Stimulation with LPS Increased Mfn2 Expression, Downregulated Autophagy, and Elevated Cell Apoptosis in Murine Splenic CD4^+^ T Cells

**Figure 2:**
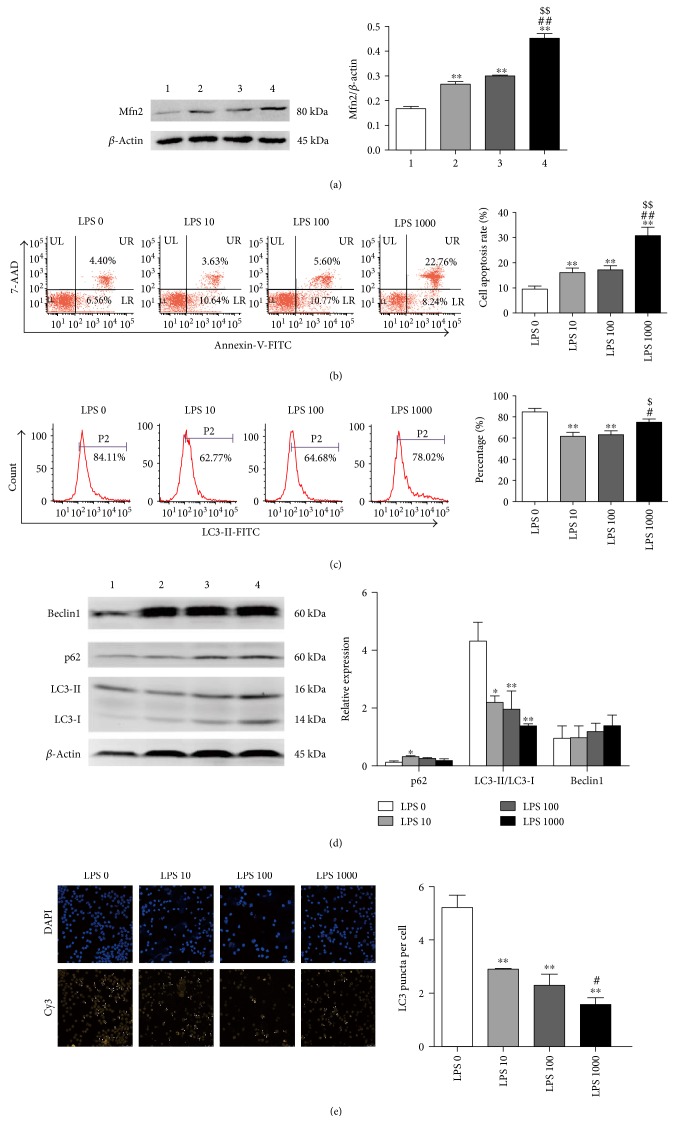
LPS stimulation increased Mfn2 expression, downregulated autophagy, and elevated apoptosis in murine splenic CD4^+^ T cells. Murine splenic CD4^+^T cells were isolated and subjected to *in vitro* culture with LPS (10, 100, and 1000 ng/ml) or PBS for 24 hours. (a) Representative immunoblots and densitometric values of Mfn2. *β*-Actin served as an internal control. (b) Representative dot plots and the percentage of Annexin-V-positive cells. (c) Representative histograms and the percentage of LC3-II-positive cells. (d) Whole cell lysate was used for immunoblot analysis of Beclin1, p62, and LC3-II expression. *β*-Actin served as an internal control for Beclin1 and p62 protein expression levels. (e) Representative images of immunofluorescence staining. DAPI and Cy3 were used to label nuclei and endogenous LC3, respectively. The arrows indicate LC3 puncta. Quantification of the average number of LC3 puncta per cell (*n* = 3 well, 3 independent experiments, and >50 cells examined per experiment). Data from three independent experiments are shown as the mean ± SD. Bar 1, the LPS 0 group; bar 2, the LPS 10 group; bar 3, the LPS 100 group; bar 4, the LPS 1000 group. ^∗^*P* < 0.05, significant difference versus the LPS 0 group. ^∗∗^*P* < 0.01, significant difference versus the LPS 0 group. ^#^*P* < 0.05, significant difference versus the LPS 10 group. ^##^*P* < 0.01, significant difference versus the LPS 10 group. ^$^*P* < 0.05, significant difference versus the LPS 100 group. ^$$^*P* < 0.01, significant difference versus the LPS 100 group.

To confirm the relationship between Mfn2 expression, autophagy level, and apoptosis in CD4^+^ T cells in sepsis, we cultured CD4^+^ T cells *in vitro* with LPS (10, 100, or 1000 ng/ml) or PBS for 24 hours. As shown in Figure 2(a), stimulation with LPS at different concentrations (10, 100, or 1000 ng/ml) resulted in enhanced expression of Mfn2, especially at 1000 ng/ml. As shown in Figure 2(b), the apoptosis rate of CD4^+^ T cells determined by Annexin-V staining was significantly increased after LPS treatment at different concentrations (10, 100, or 1000 ng/ml), especially at 1000 ng/ml.

In this experiment, flow cytometry analysis, Western blot analysis, and confocal laser scanning microscopy were used to investigate autophagy formation. Autophagic flux was evaluated by the expression of p62, which binds directly to LC3 and GABARAP family proteins (Atg orthologues) via a short LC3 interaction (LIR). As shown in Figure 2(c), the expression of LC3-II measured by flow cytometry was decreased after LPS treatment at 10 or 100 ng/ml and restored to the basal value at 1000 ng/ml. As shown in Figure 2(d), p62 expression was upregulated after LPS treatment at 10 ng/ml and displayed no difference at 100 or 1000 ng/ml; the expression of Beclin1 showed no obvious difference after culture with or without LPS; the ratio of LC3-II/LC3-I was downregulated after stimulation with LPS at various concentrations (10, 100, or 1000 ng/ml). As shown in Figure 2(e), the number of LC3 puncta per cell observed by confocal laser scanning microscopy was significantly reduced in response to LPS treatment, especially at 1000 ng/ml. Based on these data, treatment with LPS resulted in inhibition of autophagy at different concentrations (10, 100, or 1000 ng/ml).

Collectively, CD4^+^ T cells showed enhanced Mfn2 expression, autophagy deficiency, and upregulated apoptosis in response to LPS treatment.

### 3.3. Transfection Efficiency Analysis

To investigate the possible role of Mfn2 in sepsis-induced T cell apoptosis, we constructed an overexpression lentiviral vector encoding either Mfn2 (LV-Mfn2) or LV-mCherry (as a scrambled control virus) and a silencing lentiviral vector expressing sh-Mfn2 (LV-Mfn2 RNAi) or LV-RFP (as a scrambled control virus). As shown in Supplementary Figure 1a, available online at https://doi.org/10.1155/2017/4926205, the efficiency of lentiviral transfection was estimated to be greater than 90% except in the LV-Mfn2 RNAi group, whose transfection efficiency was greater than 70% because of lower proliferation of Mfn2i-transfected Jurkat T cells. As shown in Supplementary Figure 1b, LV-Mfn2-transfected cells exhibited significantly increased expression of Mfn2 compared to cells in the control group or the LV-mCherry group, whereas LV-Mfn2 RNAi-infected cells exhibited decreased expression of Mfn2 compared to cells in the control group or the LV-RFP group.

### 3.4. Overexpression of Mfn2 Inhibited Autophagy and Induced Cell Apoptosis in Jurkat T Cells

**Figure 3:**
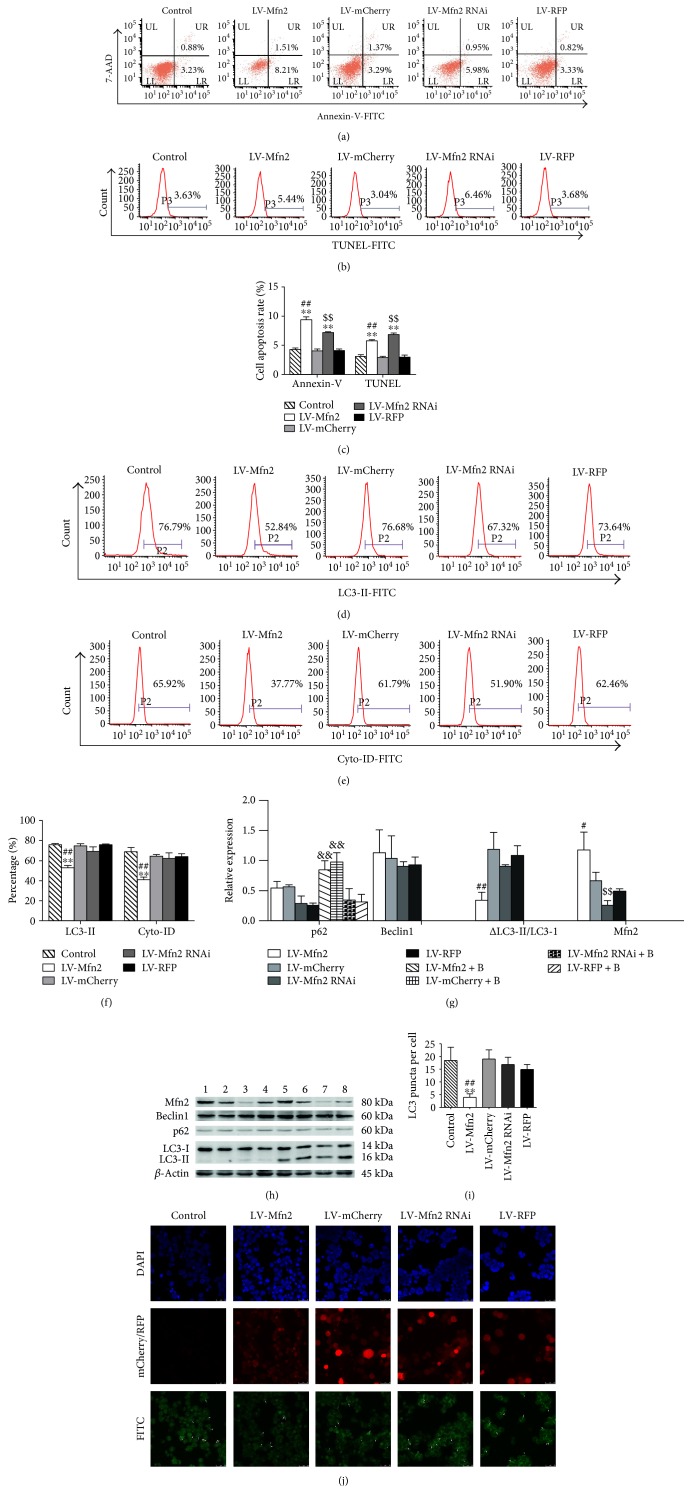
Overexpression of Mfn2 inhibited autophagy and induced cell apoptosis in Jurkat T cells. Transfected Jurkat T cells were cultured in RPMI-1640 with 10% FBS for more than 72 hours. (a) Representative dot plots of Annexin-V staining. (b) Representative histograms of TUNEL staining. (c) Percentage of apoptotic cells determined by Annexin-V or TUNEL staining. (d) Representative histograms of LC3-II-positive cells based on intracellular staining. (e) Cyto-ID was used to assess autophagosomes and autolysosomes. (f) Percentage of LC3-II- or Cyto-ID-positive cells. (g, h) Jurkat T cells were cultured with or without bafilomycin A1 (0.1 *μ*M) for 4 hours, and Western blot analysis was used to determine the expression levels of Beclin1, p62, and LC3-II protein. *β*-Actin served as an internal control for Beclin1 and p62 protein levels. (i, j) Representative images of immunofluorescence staining. DAPI and FITC were used to stain nuclei and endogenous LC3, respectively. The arrows indicate LC3 puncta. Quantification of the average number of LC3 puncta per cell (*n* = 3 well, 3 independent experiments, and >50 cells examined per experiment). The results are shown as the mean ± SD from three independent experiments. Bar 1, the LV-Mfn2 group; bar 2, the LV-mCherry group; bar 3, the LV-Mfn2 RNAi group; bar 4, the LV-RFP group; bar 5, the LV-Mfn2 + B group; bar 6, the LV-mCherry + B group; bar 7, the LV-Mfn2 RNAi + B group; bar 8, the LV-RFP + B group. Baf A1 or B: bafilomycin A1. ^∗∗^*P* < 0.01, significant difference versus the control group. ^#^*P* < 0.05, significant difference versus the LV-mCherry group. ^##^*P* < 0.01, significant difference versus the LV-mCherry group or the LV-mCherry + B group. ^$$^*P* < 0.01, significant difference versus the LV-RFP group. ^&&^*P* < 0.01, significant difference versus the corresponding group without bafilomycin A1 treatment group.

To observe the effect of Mfn2 expression on cell apoptosis, we harvested transfected Jurkat T cells and stained them with Annexin-V and TUNEL. As shown in Figures 3(a), 3(b), and 3(c), LV-Mfn2-transfected Jurkat T cells revealed an increased percentage of apoptotic cells compared to that found in the control cells or corresponding control virus-infected cells. In addition, LV-Mfn2 RNAi-transfected Jurkat T cells exhibited an increased percentage of apoptotic cells, which was consistent with our previous studies.

Next, to explore the potential immune regulation mechanism of up- or downregulated Mfn2 expression in T cell apoptosis, we determined the autophagy level. In this experiment, in addition to evaluating the expression of LC3-II, flow cytometry analysis was performed to examine autophagosomes and autolysosomes following Cyto-ID green staining. As shown in Figures 3(d), 3(e), and 3(f), LV-Mfn2-transfected cells exhibited a lower percentage of LC3-II- or Cyto-ID-positive cells versus the control group and the LV-mCherry group. By contrast, LV-Mfn2 RNAi-transfected cells showed a slight but not significant decrease in the percentage of LC3-II- or Cyto-ID-positive cells compared to that found in cells in the control group and the LV-RFP group. There was no measurable change in Beclin1 expression among groups. On the other hand, to assess autophagic flux, we treated transfected Jurkat T cells with bafilomycin A1 for 4 hours and then examined the expression of p62 and changes of LC3-II. As shown in Figures 3(g) and 3(h), LV-Mfn2- and LV-mCherry-transfected cells exhibited a significant increase in expression of p62 in response to bafilomycin A1 treatment. LV-Mfn2 RNAi- and LV-RFP-transfected cells exhibited no significant difference in expression of p62 expression upon bafilomycin A1 stimulation. To compare with LV-mCherry-transfected cells, the difference of LC3-II in LV-Mfn2-transfected cells decreased. We observed identical results when measuring autophagosome formation via immunofluorescence staining for LC3 (Figures 3(i) and 3(j)). These findings suggested that overexpression of Mfn2 negatively regulated autophagy.

These observations showed that up- or downregulation of Mfn2 expression induced T cell apoptosis via various molecular mechanisms and overexpression of Mfn2 inhibited autophagy in Jurkat T cells.

### 3.5. Pharmacological Inhibition of Autophagy Enhanced Mfn2 Overexpression-Induced Cell Apoptosis

**Figure 4:**
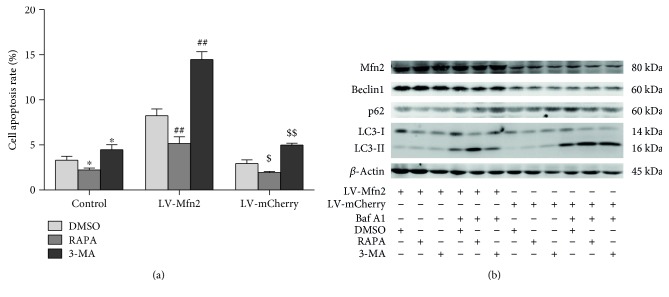
Pharmacological inhibition of autophagy enhanced Mfn2 overexpression-induced cell apoptosis. After transfection with a lentiviral vector (LV-Mfn2 or LV-mCherry), Jurkat T cells were divided into three groups and subjected to the following treatments: DMSO (vehicle control), rapamycin (100 nM, an autophagy stimulator), or 3-methyladenine (10 mM, an autophagy inhibitor). (a) The percentage of apoptotic cells based on Annexin-V staining. (b) The expression of the autophagy-related proteins Beclin1, p62, and LC3-II was measured via Western blot analysis. The results are shown as the mean ± SD. Baf A1: bafilomycin A1. RAPA: rapamycin. 3-MA: 3-methyladenine. ^∗^*P* < 0.05, significant difference versus the control + DMSO group. ^##^*P* < 0.01, significant difference versus the LV-Mfn2 + DMSO group. ^$^*P* < 0.05, significant difference versus the LV-mCherry + DMSO group. ^$$^*P* < 0.01, significant difference versus the LV-mCherry + DMSO group.

The questioned was raised whether Mfn2 overexpression-induced apoptosis is associated with its inhibition of autophagy. To answer, we first evaluated whether suppression of autophagy is sufficient to induce cell apoptosis. Jurkat T cells were treated with rapamycin (an autophagy inducer), 3-methyladenine (3-MA, an autophagy inhibitor), or DMSO (a vehicle control). As shown in Figure 4(a), the rapamycin treatment resulted in less apoptotic cells and the 3-MA treatment led to more apoptotic cells than found in the vehicle control group. We then tested whether autophagy elevation could restore the Mfn2 overexpression-induced apoptosis. Rapamycin significantly reduced Mfn2 overexpression-induced apoptosis whereas 3-MA significantly exacerbated Mfn2 overexpression-induced apoptosis. Taken together, our data indicated that suppression of autophagy contributed to the Mfn2 overexpression-induced apoptosis. To confirm the regulatory effect of rapamycin or 3-MA on autophagy, we evaluated the expression of Beclin1, p62, and LC3-II. Indeed, treatment with rapamycin resulted in increased autophagy in LV-Mfn2- or LV-mCherry-transfected cells, and treatment with 3-MA resulted in decreased autophagy (Figure 4(b)).

### 3.6. Overexpression of Mfn2 Decreased PMA/Ionomycin-, Rapamycin-, and Starvation-Induced Autophagy

**Figure 5:**
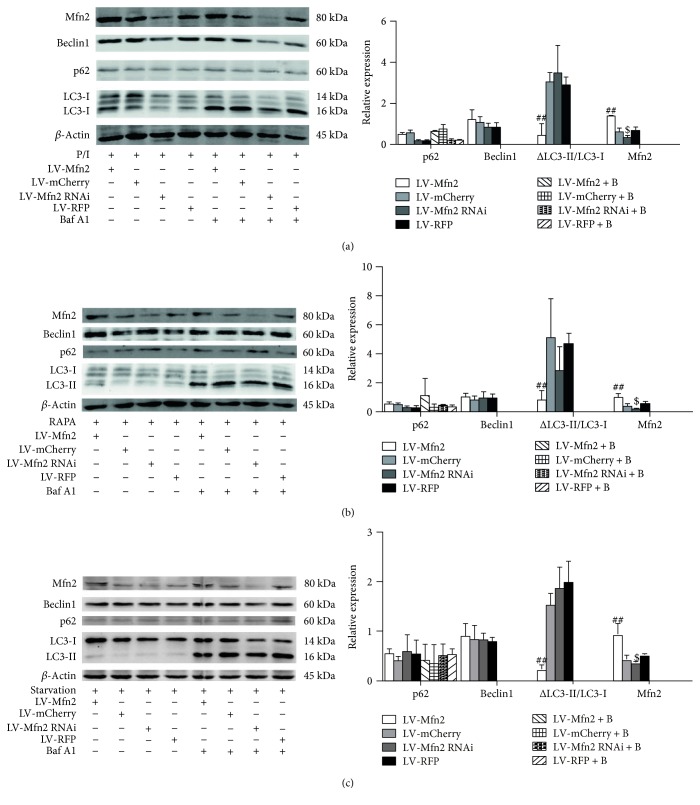
Overexpression of Mfn2 decreased PMA/ionomycin-, rapamycin-, and starvation-induced autophagy. Jurkat T cells were infected with lentiviral vector and then exposed to autophagy inducers, PMA (50 ng/ml)/ionomycin (1 *μ*M) (a), rapamycin (100 nM) (b), or starvation (low serum) (c), for the indicated intervals, followed by Western blotting to determine the expression levels of the indicated proteins. *β*-Actin served as a loading control for Beclin1 and p62 protein levels. The results of three independent experiments are shown as the mean ± SD. P/I: PMA/ionomycin. Baf A1 or B: bafilomycin A1. RAPA: rapamycin. ^##^*P* < 0.01, significant difference versus the LV-mCherry group or the LV-mCherry + B group. ^$^*P* < 0.05, significant difference versus the LV-RFP group.

We next performed a Western blot analysis to investigate the effect of up- or downregulation of Mfn2 on autophagy initiated by certain inducers, including PMA/ionomycin, rapamycin, and starvation (low serum). As shown in Figures 5(a), 5(b), and 5(c), overexpression of Mfn2 decreased PMA/ionomycin-, rapamycin-, and starvation-induced autophagy as evidenced by the decreased difference of LC3-II in the presence and absence of bafilomycin A1, but the expression of p62 and Beclin1 remained similar to that of the control. Similar to basal autophagy, knockdown of Mfn2 had no effect on PMA/ionomycin-, rapamycin-, and starvation-induced autophagy. These findings indicated that overexpression of Mfn2 inhibited PMA/ionomycin-, rapamycin-, and starvation-induced autophagy in Jurkat T cells.

## 4. Discussion

In the current study, we stated that Mfn2 acted as a negative regulatory factor of autophagy and prompted apoptosis of CD4^+^ T cells in sepsis. Accumulating evidence has shown an interaction between autophagy and apoptosis. There are many autophagy regulatory factors associated with regulation of apoptosis and vice versa, such as the interaction between Beclin1 and the BCL-2 family [17], p62 and caspase-8 [18], and Beclin1 and caspase-3 [19]. Cell death can be regulated by autophagy positively and negatively, mainly related to the nature of the death stimulus and cell type [16].

Autophagy is a catabolic process in which cellular components are degraded and recycled, and it is characterized by autophagosome formation. The autophagy process involves six principal steps: initiation, nucleation, elongation, closure, maturation, and degradation or extrusion [25]. Beclin1, LC3-II, and p62 play indispensable roles in the autophagy process. Beclin1, a component of the Beclin1-Vps34-Vps15 complex, acts as a key component in the initiation stage. LC3-II, the most reliable marker of the autophagic process at the molecular level, is associated with the elongation of step of autophagy. p62, a marker of autophagic flux, binds directly to LC3 and delivers selective autophagic cargo to be degraded. Because autophagy is a dynamic process, including autophagosome formation and autophagic degradation, LC3-II expression at some time point is not enough to indicate the autophagy level. In addition, accumulated evidence has demonstrated that LC3-II can be formed in an autophagy-independent manner [26, 27]. Thus, it is essential to assess the exact LC3-II level that is delivered to the lysosome for degradation. In this study, to evaluate autophagosome formation, we performed a Western blot analysis to measure the Beclin1 level in the absence of bafilomycin A1. In addition, flow cytometry analysis was used to evaluate the expression of membrane-associated LC3-II [28]. LC3 puncta and autophagic vacuoles observed by confocal laser scanning microscopy and TEM, respectively, were also used to evaluate autophagosome formation. Furthermore, two indicators were employed to determine autophagic flux, including p62 expression in the presence of bafilomycin A1 and the change in LC3-II expression with or without bafilomycin A1 [29].

CLP-induced peritonitis has long been used as the standard animal model for sepsis studies. Recent studies demonstrated that mitochondria dysfunction contributes to the pathology of sepsis [30, 31]. Gonzalez et al. [32] reported an imbalance in mitochondrial fusion-fission as an underlying mechanism of sepsis. However, in the present study, CD4^+^ T cells exhibited increased Mfn2 abundance after CLP, which was supported by a study showing upregulation of PGC gene (a regulator of Mfn2 gene expression) expression in a model of *Staphylococcus aureus* sepsis [33]. Meanwhile, we found an increased number of apoptotic CD4^+^ T cells after septic challenge, which was in agreement with published studies [3, 4] showing significant lymphocyte apoptosis in septic patients and animals. A series of studies have revealed that autophagy increases quickly upon septic insult in the liver, kidney, heart, and in macrophages, followed by a prolonged impaired autophagy, which is considered more important to organ dysfunction [34–38]. In addition, increased autophagy can protect organs from sepsis-induced injury [39–43]. Nevertheless, in the present study, we demonstrated that autophagy was inhibited at 24 hours and subsequently increased at 72 hours in CD4^+^ T cells after CLP. We think that the contradiction between results from previous studies and the present experiment might be due to the different time points, tissue type, and severity of sepsis. Previous studies focused on the early 24-hour time point, whereas our present study examined the 24- and 72-hour range. The former studies evaluated autophagy in organ tissue or in macrophages, whereas our study examined autophagy in CD4^+^ T cells. In addition, there is no single standard for the severity of sepsis. Lin et al. [22] showed autophagy deficiency in T cells in sepsis, which was consistent with our results. The results of bidirectional regulation of autophagy suggest possible dual roles in CD4^+^ T cells after CLP, which have been shown in LPS-induced mDPC6T cells [44]. Based on these data, it might be concluded that CD4^+^ T cells exhibit increased Mfn2 expression, decreased autophagy, and elevated cell apoptosis 24 hours after CLP.

In the *in vitro* experiment, we employed LPS, a major endotoxin in sepsis extracted from the wall of gram-negative cells, to mimic a sepsis state. In this study, we demonstrated increased Mfn2 abundance, autophagy deficiency, and upregulated apoptosis in CD4^+^ T cells upon LPS stimulation at different concentrations (10, 100, and 1000 ng/ml). However, the data obtained from flow cytometry to detect LC3-II were different from the results from the Western blot analysis and confocal laser scanning microscopy. It is highly probable that we ignore the average fluorescence intensity in the data showing the percentage of LC3-II-positive cells. Given the similar trend of the *in vivo* and *in vitro* studies, we hypothesize that Mfn2 might be a regulatory factor of CD4^+^ T cell apoptosis in sepsis and autophagy deficiency might be the molecular mechanism.

To verify our hypothesis, LV-Mfn2 lentiviral vectors were constructed and used to transfect Jurkat T cells to upregulate Mfn2 expression to reveal the underlying mechanism of T cell apoptosis induced by Mfn2. Nevertheless, our previous studies illustrated that decreased Mfn2 level was also associated with apoptosis and decreased proliferation in T lymphocytes treated with HMGB1 [12, 13]. So LV-Mfn2 RNAi lentiviral vectors were also constructed and used to transfect Jurkat T cells to downregulate Mfn2 expression in the present study. In the current study, both overexpression of Mfn2 and silencing of Mfn2 resulted in significantly increased cell apoptosis, which was consistent with findings from previous studies [8, 9, 11, 45, 46]. These data imply that a balance of Mfn2 expression maintains cellular homeostasis, whereas an imbalance leads to cell apoptosis. Two different molecular pathways may be involved in these two events. In our previous studies, we revealed that decreased expression of mfn2 was involved in HMGB1-mediated apoptosis of CD4+ T cells and partly through Ca^2+^-NFAT signaling pathway.

To investigate the potential mechanism, we determined the autophagy level. LV-Mfn2-transfected cells exhibited decreased autophagy; by contrast, LV-Mfn2 RNAi-transfected cells exhibited no detectable change in autophagy. Unexpectedly, the Beclin1 expression showed no detectable change, nor did the p62 level, suggesting that the autophagy was Beclin1 independent. The p62 level exhibited no significant difference for two reasons. Firstly, the autophagy was p62 independent; secondly, quantification of p62 levels serves as an indirect measurement of the efficient removal of the cargo. The guidelines published by Klionsky et al. have already noted some limitations in analyzing p62 by WB, that is, its protein solubility, when specific lysis buffers are used, without any consensus of a perfect one. However, Zhao et al. [24] reported that knockdown of Mfn2 disturbed autophagosome-lysosome fusion in cardiomyocytes. Another study by Ding et al. [47] showed that silencing Mfn2 impaired autophagic degradation. The difference in transfection efficiency or cell type could account for the discrepancy. Downregulation of Mfn2 can lead to mitochondrial fragmentation, mitochondrial dysfunction [11, 48], and endoplasmic reticulum stress [49], which may be the mechanism underlying Mfn2 silencing-induced apoptosis. Taken together, these findings indicate that autophagy deficiency may be involved in Mfn2 overexpression-induced cell apoptosis but not the mechanism of cell apoptosis induced by downregulated Mfn2 expression which is consistent with our previous studies [12, 13]. Additionally, 3-methyladenine, an autophagy inhibitor, worsens the apoptosis of LV-Mfn2-transfected cells, whereas rapamycin, an autophagy stimulator, reduced the apoptosis of LV-Mfn2-transfected cells, suggesting that autophagy inhibition is the mediator involved in Mfn2 overexpression-induced cell apoptosis. This notion is supported by the study of Gump et al. [16], showing that autophagy acts as a proapoptosis role after Fas-ligand stimulation by degrading Fap-1 selectively in type I cells, whereas, autophagy plays an antiapoptotic role in type II cells (Jurkat T cells) or upon TRAIL treatment in type I and type II cells. Rapamycin, an mTOR inhibitor, restored the autophagy deficiency induced by Mfn2 overexpression, suggesting that Mfn2 acts upstream of mTOR.

After investigating the effect of Mfn2 expression on basal autophagy, another question regarding whether Mfn2 expression affected induced autophagy was raised. To answer this question, PMA/ionomycin, rapamycin, and starvation were applied to induce autophagy. Similar to basal autophagy, overexpression of Mfn2 resulted in impaired autophagy. Whereas, silencing of Mfn2 had no effect on PMA/ionomycin-, rapamycin-, or starvation-induced autophagy, similar to the results observed with basal autophagy.

There are some limitations in this study. The transfection experiment was undertaken in Jurkat T cells because there is not an effective transfection system to study autophagy in primary CD4^+^ T cells. Transient transfection increases autophagy, and lentiviral transfection results in a low transfection efficiency and low cell viability of primary CD4^+^ T cells. Further investigation should be undertaken to explore the molecular pathway underlying negative regulation of autophagy by Mfn2. In addition, experiments to evaluate the precise mechanism underlying the apoptosis induced by Mfn2 silencing should be performed.

Despite the many advances in therapeutic strategies, such as extensive methods for supportive care and fluid resuscitation, unfortunately, the mortality of severely septic patients is still high. Hence, it is urgent to find a more effective therapeutic approach to treat septic patients. This study reveals an alternative mechanism in CD4^+^ T cell apoptosis in sepsis and provides insight for the development of novel interventional strategies to prevent apoptosis of CD4^+^ T cells in sepsis. In addition, this study presents a potential mechanism contributing to downregulation of autophagic activity in the setting of sepsis.

## Supplementary Material

Supplementary Figure 1 Transfection efficiency analysis Jurkat T cells were transfected with a lentiviral vectorLV-Mfn2, LV-mCherry (over-expression scramble control), LV-Mfn2RNAi or LV-RFP (slience scramble control) at MOI=50. (a) After 72hours, cells were observed under a fluorescence microscope. (b) The expression of protein Mfn2 was measured by Western blot analysis. β-actin was used as a loading control. Results of three independent experiments were shown as the mean±SD in the bar graph. Bar 1, the control group; Bar 2, the LV-Mfn2 group; Bar 3, the LV-mCherry group; Bar 4, the LV-Mfn2RNAi group; Bar 5, the LV-RFP group. ∗P<0.05, significant difference vs. the control group. ∗∗P<0.01, significant difference vs. the control group. ##P<0.01, significant difference vs. the LV-mCherry group. $P<0.05, significant difference vs. the LV-RFP group.
