# Supplementary material for: Mitofusin 2 Promotes Apoptosis of CD4+ T Cells by Inhibiting Autophagy in Sepsis
Source: Mediators Inflamm. 2017 Nov 19;2017:4926205. doi: 10.1155/2017/4926205 (PMC5735308; doi:10.1155/2017/4926205)

Supplementary Figure 1 Transfection efficiency analysis Jurkat T cells were transfected with a lentiviral vector LV-Mfn2, LV-mCherry (over-expression scramble control), LV-Mfn2 RNAi or LV-RFP (slience scramble control) at MOI=50. (a) After 72 hours, cells were observed under a fluorescence microscope. (b) The expression of protein Mfn2 was measured by Western blot analysis. β-actin was used as a loading control. Results of three independent experiments were shown as the mean±SD in the bar graph. Bar 1, the control group; Bar 2, the LV-Mfn2 group; Bar 3, the LV-mCherry group; Bar 4, the LV-Mfn2RNAi group; Bar 5, the LV-RFP group. *P<0.05, significant difference vs. the control group. **P<0.01, significant difference vs. the control group. ##P<0.01, significant difference vs. the LV-mCherry group. $P<0.05, significant difference vs. the LV-RFP group.

Supplementary Figure 1

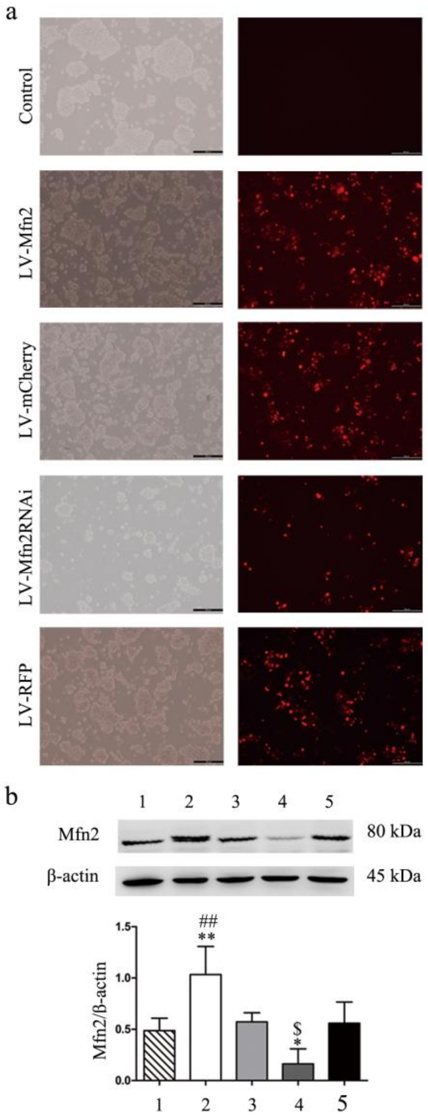

Supplement: Supplementary file 1 — Supplementary Figure 1 Transfection efficiency analysis Jurkat T cells were transfected with a lentiviral vectorLV-Mfn2, LV-mCherry (over-expression scramble control), LV-Mfn2RNAi or LV-RFP (slience scramble control) at MOI=50. (a) After 72hours, cells were observed under a fluorescence microscope. (b) The expression of protein Mfn2 was measured by Western blot analysis. β-actin was used as a loading control. Results of three independent experiments were shown as the mean±SD in the bar graph. Bar 1, the control group; Bar 2, the LV-Mfn2 group; Bar 3, the LV-mCherry group; Bar 4, the LV-Mfn2RNAi group; Bar 5, the LV-RFP group. ∗P<0.05, significant difference vs. the control group. ∗∗P<0.01, significant difference vs. the control group. ##P<0.01, significant difference vs. the LV-mCherry group. $P<0.05, significant difference vs. the LV-RFP group. [file 4926205.f1.pdf]
